# New Insights Into the Human Dopamine Transporter: Structure, Function, and Therapeutic Potential

**DOI:** 10.1002/mco2.70187

**Published:** 2025-04-14

**Authors:** Qi Weng, Qi Wu, Quan Zheng

**Affiliations:** ^1^ Department of Pharmacy The Quzhou Affiliated Hospital of Wenzhou Medical University Quzhou People's Hospital Quzhou China; ^2^ Department of Medical Oncology and Core Facility The Quzhou Affiliated Hospital of Wenzhou Medical University Quzhou People's Hospital Quzhou China

1

Recently, three breakthrough studies on the structure and function of the human dopamine transporter (hDAT) were published consecutively in *Nature* [1, 2, 3]. Researchers used cryo‐electron microscopy to resolve high‐resolution structures of hDAT bound to various ligands, including dopamine (DA), methylphenidate (MPH), β‐CFT, GBR12909, MRS7292, benztropine, and cocaine. The resolution of these structures revealed the detailed molecular mechanism of hDAT in DA reuptake and inhibition, providing important information to guide the development of drugs for the treatment of DA‐related diseases.

DA is an important neurotransmitter that is involved in regulating a variety of functions in the brain, including cognition, movement, emotion, and reward [4]. The dopamine transporter (DAT) is located in the presynaptic membrane of dopaminergic neurons and is responsible for the reuptake of DA in the synaptic gap, inhibition of DA signaling, and maintenance of central nervous system DA homeostasis. When hDAT malfunctions, it may lead to abnormal DA levels, which are associated with a variety of disorders such as attention deficit hyperactivity disorder (ADHD), depression, bipolar disorder, Parkinson's disease, and addictive behaviors [5]. Although scientists have been studying DAT for decades, much remains unknown about the structure, conformational transitions, and specific postures of hDAT for drug binding. Therefore, an in‐depth study of the structure and function of hDAT is extremely important for the development of new drugs for the treatment of DA‐related diseases.

Li et al. [1] observed three different conformational changes of hDAT during DA transport by cryo‐electron microscopy, including the outward‐open state, the closed state, and the inward‐open state (Figure 1). It is evident that Na^+^ and Cl^−^ play key roles in the reuptake of DA, with Na^+^ interacting with residues N82, N353, D421, and S422, while Cl^−^ binds to Y102, Q317, S321, and S357. These ions form hydrogen bonds and charge interactions that stabilize DA binding in hDAT and facilitate its transport from extracellular to intracellular compartments. Notably, hDAT has fewer water molecules in the DA binding site, a phenomenon that may affect DA binding and transporter function. This creates a more hydrophobic environment, enhancing DA binding stability but reducing flexibility and binding/dissociation rates. Additionally, fewer water molecules reduce hydrogen‐bonding networks, increasing reliance on direct interactions between amino acids and DA. This raises the specificity of the binding but also increases the sensitivity to structural changes.

**FIGURE 1:**
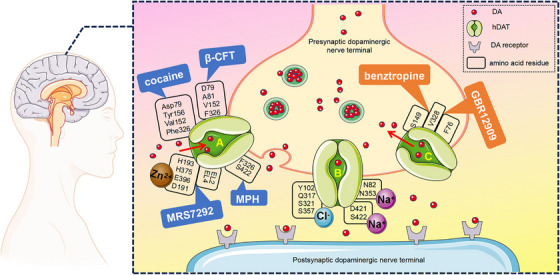
Molecular mechanisms of the human dopamine transporter (hDAT) in dopamine (DA) reuptake and inhibition. hDAT achieves DA transport by altering the conformation of hDAT with three main conformational states—(A) outward‐open state (open to the outside in preparation for DA binding), (B) closed state (after DA binding, hDAT closes to protect DA from the external environment), (C) the inward open state (DA is released into the interior of the cell). Two Na^+^ and one Cl^−^ binding sites were observed in the closed state of hDAT. MPH, β‐CFT, and cocaine binding to hDAT in the binding pocket towards the outer opening of the cell stabilized hDAT in the outwardly open state, thereby inhibiting DA transport. However, GBR12909 and benztropine binding to hDAT in the binding pocket towards the inner cell opening stabilizes DAT in the inner open state, thereby inhibiting DA transport. In addition, MRS7292 and Zn^2+^ stabilize hDAT in the outward‐open state and inhibit DA transport through synergistic cooperation at a newly discovered allosteric site.

Meanwhile, Srivastava et al. [2] also successfully resolved the three‐dimensional structure of hDAT in the binding state with the cocaine analog competitive inhibitor β‐CFT, the noncompetitive inhibitor MRS7292, and Zn^2+^. These high‐resolution structural images showed that hDAT presents an outwardly open conformation under different inhibition conditions. Specifically, β‐CFT occupied the central binding site of hDAT and interacted with key residues such as D79, A81, V152, and F326, effectively stabilizing its outward open state. Notably, the noncompetitive inhibitor MRS7292 bound to a previously understudied variant site, and this binding inhibited the closure of the outward gate. In addition, Zn^2+^ in a tetra‐coordinated form with four amino acid residues (H193, H375, E396, and D191) bridged the EL2 and EL4 extracellular loop regions, which further consolidated the externally oriented conformation of hDAT and provided new insights into how Zn^2+^ inhibits its transport activities.

Li et al. [1] focused on the drug MPH, which is used to treat ADHD, and found that it binds differently to DA. MPH occupied the binding pocket of hDAT on the extracellular side through a subtle mechanism, and this occupancy stabilized the outwardly open state of hDAT. Furthermore, MPH achieved this stabilizing effect by forming hydrophobic interactions with amino acid residues in the transmembrane region of hDAT (e.g., F326 and S422). This distinctive binding modality effectively blocked its ability to turn to the closed state, thereby inhibiting the transporter activity of hDAT. This inhibition leads directly to an elevated concentration of DA in the synaptic gap, providing patients with significant benefits such as a reduction in hyperactive and impulsive behaviors, as well as an enhanced ability to concentrate.

However, excessive inhibition of hDAT may also trigger a range of side effects, including the risk of drug addiction, which has become a focus of concern within the medical community. Recently, Nielsen et al. [3] have revealed for the first time the high‐resolution structure of the hDAT‐cocaine complex using high‐precision cryo‐electron microscopy. Further studies have shown that the cocaine molecule stabilizes the outwardly open state of hDAT by forming hydrogen bonds and hydrophobic interactions with specific amino acid residues of hDAT (Asp79, Tyr156, Val152, and Phe326). This conformational change may explain how cocaine increases DA release, leading to its addictive and euphoric effects.

In contrast to the binding pattern of cocaine and MPH, GBR12909 and benztropine stabilize hDAT in an inward‐opening conformation. These drugs may diminish the stimulatory effects of addictive drugs on brain reward circuits by modulating the functional state of hDAT and reducing the abnormal accumulation of DA in the synaptic gap. Compared to benztropine, GBR12909 exhibited a greater affinity. Despite the partial overlap of their binding sites, GBR12909 interacted more significantly with the transmembrane structural domain of hDAT, resulting in more effective inhibition of DA reuptake. Moreover, the long hydrophobic chain and piperazine ring of GBR12909 can establish stronger hydrophobic interactions and π–π stacking with the binding pocket of hDAT. In contrast, the bicyclic structure of benztropine restricts its flexibility within the binding pocket, thereby decreasing its affinity. In addition, the researchers found that specific amino acid residues (S149, V328, and F76) on DAT are critical for the binding of GBR12909 and benztropine, which provides new targets for the design of more efficient anti‐addiction drugs in the future.

In summary, the three recent breakthrough studies are not only landmarks in the field of structural biology but also show great potential for application in clinical therapeutic areas. These studies provided important ideas and targets for the development of novel drugs. By targeting the hydrophobic pockets or allosteric sites of hDAT, inhibitors with high selectivity and low addictive potential can be designed to precisely regulate the conformation of hDAT, which in turn affects the reuptake of DA. In addition, the study revealed the critical role of Zn^2^⁺ in regulating hDAT function, which laid the foundation for the development of novel drugs based on metal ion modulation. Future studies will explore the link between the structure and function of hDAT in greater depth. For example, we could study the mechanism of action of hDAT in specific diseases and how drugs affect its function. This will help us to more fully understand the role of DA transporter proteins in neurodegenerative diseases, drug addiction, and other related disorders and provide a scientific basis for developing new therapeutic strategies.

## Author Contributions

Quan Zheng conceived the manuscript. Qi Weng wrote the manuscript. Qi Wu prepared the figure. Qi Weng and Quan Zheng proofread and revised the manuscript. All authors have read and approved the final manuscript.

## Ethics Statement

The authors have nothing to report.

## Conflicts of Interest

The authors declare no conflicts of interest.

## Data Availability

The authors have nothing to report.

## References

[1] Y. Li , X. Wang , Y. Meng , et al., “Dopamine Reuptake and Inhibitory Mechanisms in Human Dopamine Transporter,” Nature 632 (2024): 686–694. 39112701 10.1038/s41586-024-07796-0

[2] D. K. Srivastava , V. Navratna , D. K. Tosh , et al., “Structure of the Human Dopamine Transporter and Mechanisms of Inhibition,” Nature 632 (2024): 672–677. 39112705 10.1038/s41586-024-07739-9 PMC11324517

[3] J. C. Nielsen , K. Salomon , I. E. Kalenderoglou , et al., “Structure of the Human Dopamine Transporter in Complex With Cocaine,” Nature 632 (2024): 678–685. 39112703 10.1038/s41586-024-07804-3

[4] B. Channer , S. M. Matt , E. A. Nickoloff‐Bybel , et al., “Dopamine, Immunity, and Disease,” Pharmacological Reviews 75, no. 1 (2023): 62–158. 36757901 10.1124/pharmrev.122.000618 PMC9832385

[5] M. E. A. Reith , S. Kortagere , C. E. Wiers , et al., “The Dopamine Transporter Gene *SLC6A3*: Multidisease Risks,” Molecular Psychiatry 27, no. 2 (2022): 1031–1046. 34650206 10.1038/s41380-021-01341-5 PMC9008071

